# Cutaneous Leishmaniasis in Pakistan: a neglected disease needing one health strategy

**DOI:** 10.1186/s12879-021-06327-w

**Published:** 2021-06-30

**Authors:** Behzad Kayani, Shakera Sadiq, Hamad Bin Rashid, Naseer Ahmed, Altaf Mahmood, Muhammad Shakeel Khaliq, Rubab Maqsood, Haroon Rashid, Saima Hasan, Muhammad Hassan Mushtaq, Ubaid-ur-Rehman Zia, Mamoona Chaudhry

**Affiliations:** 1 Department of Epidemiology and Public Health, University of Veterinary and Animal Sciences, Lahore, Pakistan; 2 Department of Clinical Medicine and Surgery, University of Veterinary and Animal Sciences, Lahore, Pakistan; 3 District Health Development Center, Jhelum, Pakistan; 4 Directorate of Animal Disease, Diagnostic, Reporting, and Surveillance, Livestock and Dairy Development Department, Government of Punjab, Lahore, Pakistan; 5 Department of Microbiology, Faculty of Veterinary Sciences, Islamia University Bahawalpur, Bahawalpur, Pakistan

**Keywords:** Leishmaniasis, Sand-fly, Cutaneous, Vector-borne, Risk factors, Case-control, Pakistan, Zoonosis

## Abstract

**Background:**

Cutaneous Leishmaniasis (CL) is a neglected tropical disease, which mainly affects poor communities. It is one of the major vector-borne disease and endemic in Pakistan.

**Methods:**

A case-control study to evaluate potential risk factors of human-CL was conducted in Khewra region, District Jhelum, Pakistan from January–April 2014. Case data about 90 cases registered during October 2012 to November 2013 was retrieved from Municipal Hospital. Controls were matched (1,1 ratio) on the date of registration with cases from same hospital. Both cases and controls were invited to participate and data was collected in a face-to-face interview. A prospective study of canine leishmaniasis (canine-CL) was also conducted at Civil Veterinary Hospital in the same area. Suspected dogs with skin ulceration signs were included in the study and blood samples were collected. Statistical analyses were conducted to determine association between various parameters and outcome of interest.

**Results:**

The ages of cases ranged from 1 to 76 years (median = 15 years) and proved to be protective factor i.e. increase in each year in age reduced the likelihood of being infected with human-CL [Odds Ratio (OR) = 0.4, 95% Confidence Interval (CI) = 0.25–0.76]. People sleeping outsides in an open area were more likely to become a case (OR = 8.7, 95% CI = 2.90–26.37) than a control. Poor sanitary condition inside the house (OR = 3.3, 95% CI 1.03–10.56) and presence of other animals in house (livestock, poultry) (OR = 3.6, 95% CI = 1.07–12.12) also identified as risk factors of high significance. The proportion of positive dogs with canine-CL was 21.05% and was significantly associated with human-CL cases in the same area (*p* < 0.05).

**Conclusions:**

We concluded that adopting self-protections measures against sand-fly, and maintaining good hygiene may lower the risk of human-CL. One-Health Strategy is suggested to control leishmaniasis in human and dog population.

**Supplementary Information:**

The online version contains supplementary material available at 10.1186/s12879-021-06327-w.

## Background

Cutaneous Leishmaniasis (CL) is a parasitic disease transmitted via the bite of female sand-flies belonging to the genera *Phlebotomus* in the Old World and *Lutzomyia* in the New World. It is a skin disease ranging from self-healing lesions to single or large skin ulcers and is caused by protozoan parasites of the genus *Leishmania* [1]*. Leishmania major* is a main cause of CL in humans in an area that stretches from India through Central Asia, the Middle East, to North and West Africa [2, 3].

The epidemiology of leishmaniases is dynamic and the conditions of transmission are continually changing depending on change in environment, demography, human behavior, socioeconomic status, and immunogenic profile of affected human populations [2, 4]. Among the most important zoonotic diseases, leishmaniasis is a major concern for public health [5]. In terms of burden of diseases, it is estimated to be the third most important vector-borne disease. Despite this fact, it is one of the “neglected diseases”. The tropical and sub-tropical parts of the world are endemic with leishmaniasis [3]. The disease occurs in 88 countries of the world with 70 being endemic. Afghanistan, Algeria, Brazil, Pakistan, Peru, Saudi Arabia, and Syria are the countries where 90% of the cases occur [6]. According to an estimate, 1.3 million new cases and 20,000 to 30,000 deaths occur annually [5]. The disease is disfiguring skin affliction as reported by U.S Centre of Disease Control and Prevention (CDC). Leishmaniasis can be divided into two forms based on epidemiology of disease: zoonotic which includes animal reservoir hosts in the transmission cycle of the disease, and anthroponotic, in which humans are considered to be the sole source of infection for the sand-fly vector [7].

In Pakistan, leishmaniasis has been reported in human and animal population [8, 9]. Human-CL is endemic in several parts of Pakistan and is the second most prevalent vector-borne disease in the country after malaria [10]. There are 37 out of 70 species of the sand-fly inhabitant in Pakistan, which can transmit disease to healthy hosts [11]. Endemic areas of disease in Pakistan include areas of Baluchistan, Interior Sindh, South Punjab and Khyber Pakhtunkhwa [11–14]. Currently, the progression of the disease is a public health issue and represents a challenge for health professionals. Epidemiological studies might help planning for effective strategies to control human-CL. Several factors such as climatic and environmental changes, the movement or migration of infected people, animal reservoirs and female infected sand-flies play important role in the transmission of leishmaniasis [15].

In Pakistan, cases of human-CL have been reported from different districts of Punjab province, however, data is scant about the identification of risk factors specifically in District Jhelum. Few studies have been carried out in Baluchistan, Khyber Pakhtunkhwa and Azad Kashmir [11, 12, 16]. In the present study, we aimed to quantify risk factors associated with human-CL in Khewra region of District Jhelum, Pakistan, with an objective to inform policy makers for evidence-based disease control recommendations to prevent future outbreaks. To study the presence of zoonotic risk of human-CL, we also conducted a prospective study in dogs, suspected for canine leishmaniasis (canine-CL) in the same geographical area.

## Methods

### Study area

**Fig. 1:**
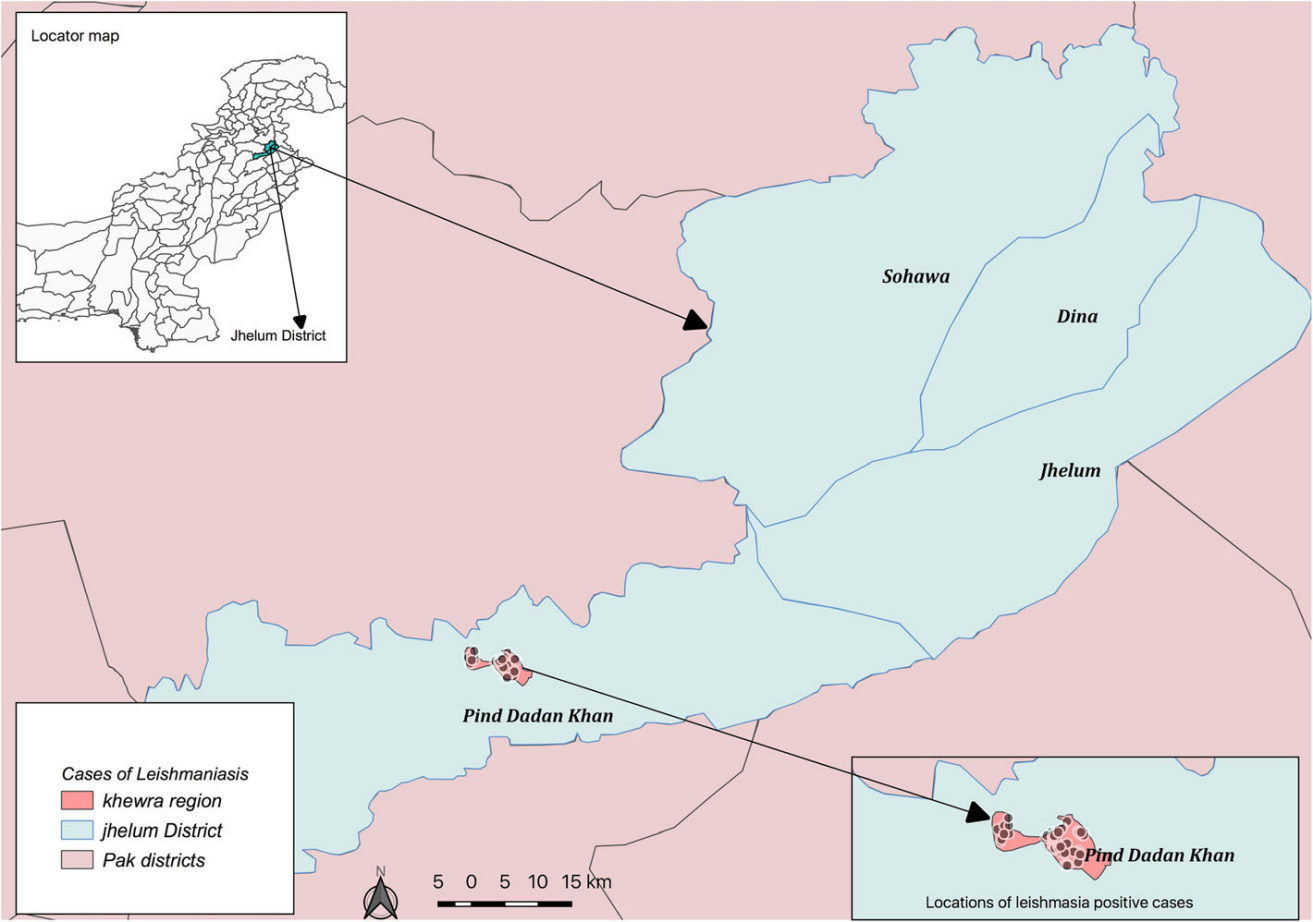
Map showing study area in District Jhelum, Pakistan

The study was conducted in Khewra region, Tehsil Pind Dadan Khan, District Jhelum (Fig. 1). The district is administratively divided into four tehsils namely Jhelum, Dina, Sohawa and Pind Dadan Khan. Khewra region is divided into two union councils: Khewra no.1 and Khewra no.2 with a population of around 35,000 [17]. The area is surrounded by the famous Salt Range. It is located at 32°38′ 60″ N 73° 1′ 0″ E. Khewra City is also known as “The Kingdom of Salt” because of its rock salt, which is 98% pure and natural source of salt in Pakistan. Khewra Salt Mine is the second largest salt mine in the world [18, 19]. Previously several outbreaks of human-CL in local population have been reported from this area between 2012 and 2013 [20].

### Study design

#### Case-control study in human

A case-control study was designed to evaluate the risk factors associated with human-CL between January to April 2014 in residents of Khewra region, District Jhelum. Patient records from outpatient and inpatient clinics of Municipal Hospital, Pind Dadan Khan, District Jhelum, were retrieved and reviewed for case selection.

### Definition of case and control

Human-CL positive cases were diagnosed by medical physicians at the Municipal Hospital. A case was defined as a person having at least one leishmania lesion (presence of a skin ulcer with typical raised edges and depressed centre or a skin plaque-a circumscribed, nodular or palpable skin lesion) and/or a typical scar (a typical CL scar develop when a papule appears after biting of sand-fly, which may enlarge to become an indolent ulcerated nodule or plaque, and after self-healing of the plague, a depressed scar is left on skin) [21]. The trained medical officers used clinical diagnosis (leishmania lesion or/and CL scar) followed by confirmatory microscopy (impression smear) to confirm leishmaniasis. Case patients were visited in their house after getting their information from hospital records.

Human-CL negative controls were selected from the same hospital registered on the same day with different complain (visiting hospital to seek treatment for other diseases like trauma, accidents, surgeries, respiratory infections etc.) and had no typical skin lesions (ulcer, plaque, wound or scar) upon inquiry by investigation team.

All participants consented to participate in the study. Institutional Committee for Biomedical Research at University of Veterinary and Animal Sciences, Lahore, Pakistan (Letter no. 077/IRC/BMR) approved the study design. Permission to conduct study was obtained from Municipal Hospital authorities. Anonymity and confidentiality of patient data were assured.

### Sample size calculation

A sample size of 180 individual (90 cases and 90 controls) was determined to give the study, 80% power at 5% significance to detect an odds ratio (OR) of > 2 for an exposure of human-CL in 30% of controls [22]. From the list of confirmed cases, 90 cases of human-CL were selected randomly and matched with 90 confirmed control on the date of registration in the hospital with a case–control ratio of 1:1. Sample size was calculated using epiR package version 1.0–14 [23] in R software.

### Enrollment of cases and controls

We contacted and enrolled 180 participants for the case-control study (90 cases who could be reached during the study period were selected from the hospital records based on the case definition). Each enrolled case was matched with a hospital-based control (90 control) by the date of registration to the hospital.

### Data collection and analyses

A predesigned questionnaire (Supplementary Material) was administered to cases and controls through face-to-face interviews. Questionnaire comprised of two sections namely general information and exposure information and closed question about potential risk factors were asked. Information about age, sex and different socio-demographic factors of cases and controls was collected.

The data was compiled by making a database in Microsoft Excel. R software version 2.14.0 [24] was used to statistically analyze the data. Simple proportions, means and medians were calculated for categorical data and continuous data respectively. To identify biologically plausible risk factors associated with the human-CL, conditional logistic regression was conducted by using survival package (version, 2.36.10) in R software, which effectively performs a Mantel-Haenszel matched-pair analysis [25]. Variables with *p* < 0.25 in the univariable analyses were consequently included in multivariable analysis for final model building. To develop the final model, multivariable logistic regression was conducted using forward elimination method, starting with most significant factors having lowest *p-value* in the univariable analysis to determine independent risk factors [26]. Odds ratios and 95% confidence intervals (95% CI) were computed for significant risk factors to measure strength of association. All statistical tests were performed at a significance level of 0.05.

QGIS version 2.14.3 (available at https://www.qgis.org/en/site/forusers/download.html#) was used to visualize the spatial distributions of cases in Khewra region, District Jhelum.

### Prospective study in dogs

A prospective study of canine-CL in pet dogs attending Civil Veterinary Hospital from Khewra region, was conducted from January–April 2014. Owners of all suspected dogs with skin lesions (dermatitis, alopecia, cutaneous ulcerations, weight loss, ocular or nasal lesions) [27, 28] attending the government veterinary hospital were requested to participate in study. Only those dogs were included whose owners consented to participate in the study. Peripheral blood samples were collected by a trained veterinarian from suspected dogs and thin dry smears were made using leishman’s stain. The amastigotes of *Leishmania* were detected by using a compound microscope [29].

### Data analysis

Data was statistically analyzed by using R software version 2.14.0 [24]. Chi square test was used to asses any association between canine-CL, area, and presence of any positive human-CL case. Proportion of canine-CL was calculated.

## Results

The human-CL cases enrolled in current study were registered in Municipal Hospital, Pind Dadan Khan, District Jhelum from October 2012 to November 2013. All of them visited hospital after the development of the lesions. Therefore, the date of the sand-fly bite was not accurately known by the cases and the time of exposure to human-CL could not be specified.

### Demographic characteristics of cases

**Fig. 2:**
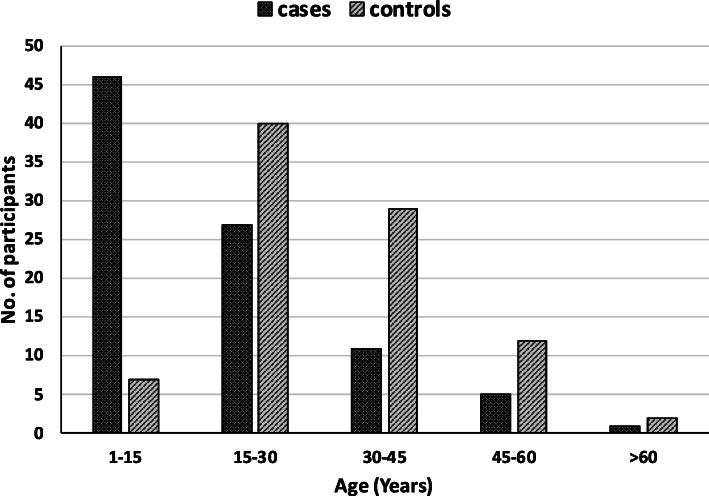
Age of study participants

**Fig. 3:**
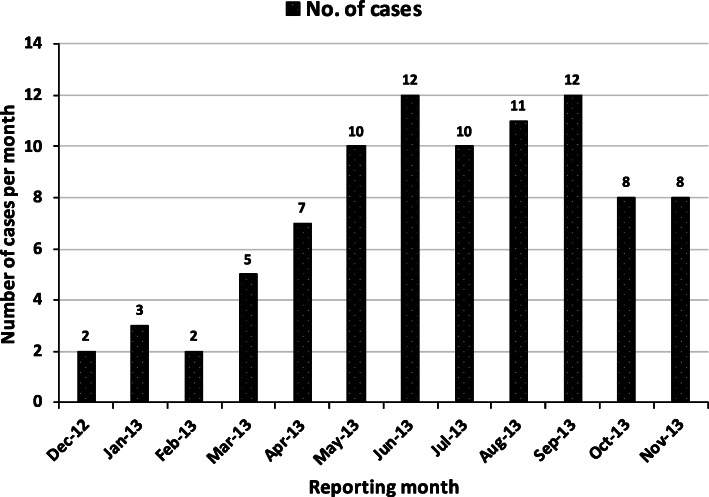
Distribution of reported cases of human-CL per month

**Fig. 4:**
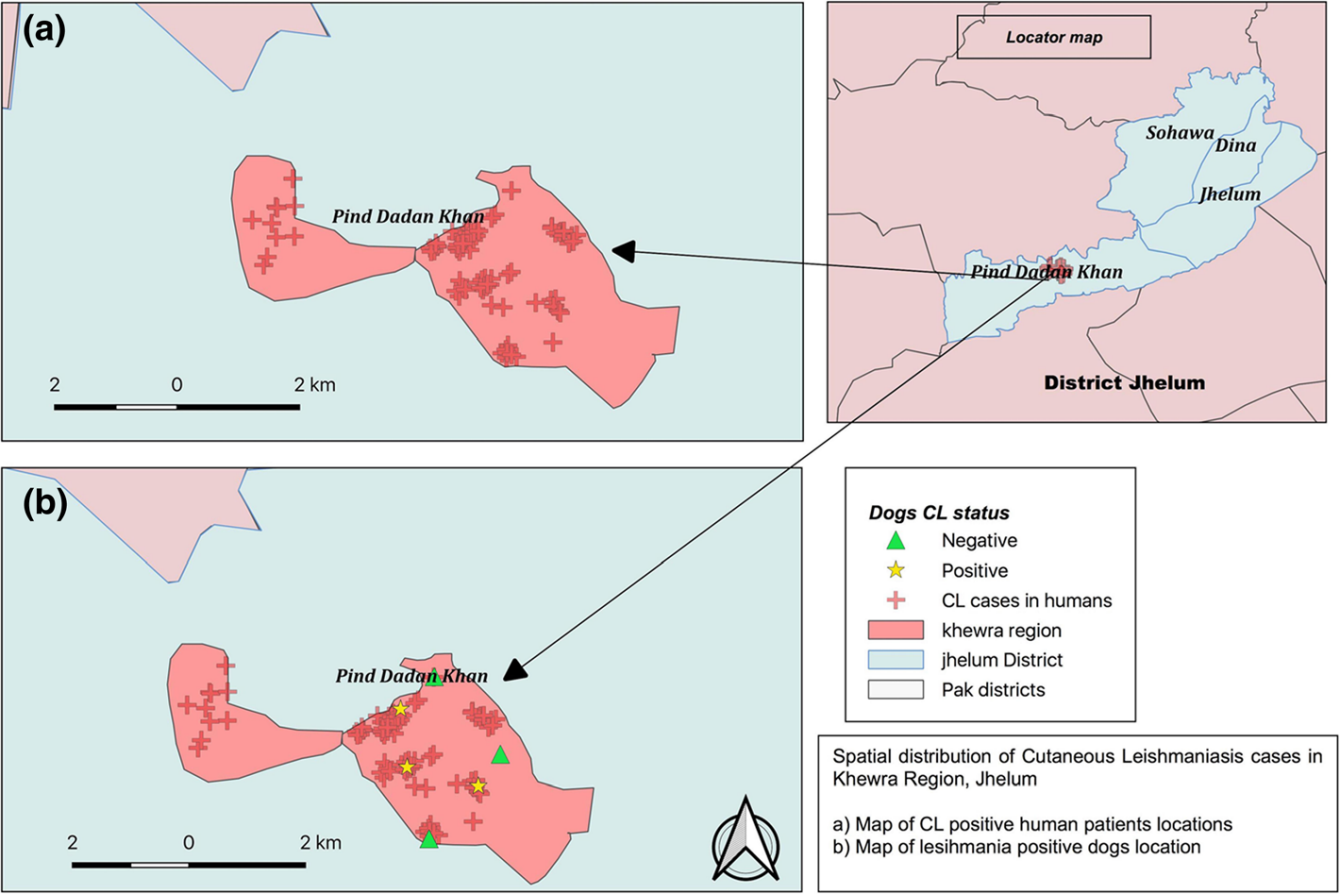
Locations of enrolled cases of human-CL (a) and canine-CL (b) in Khewra Region, District Jhelum

**Fig. 5:**
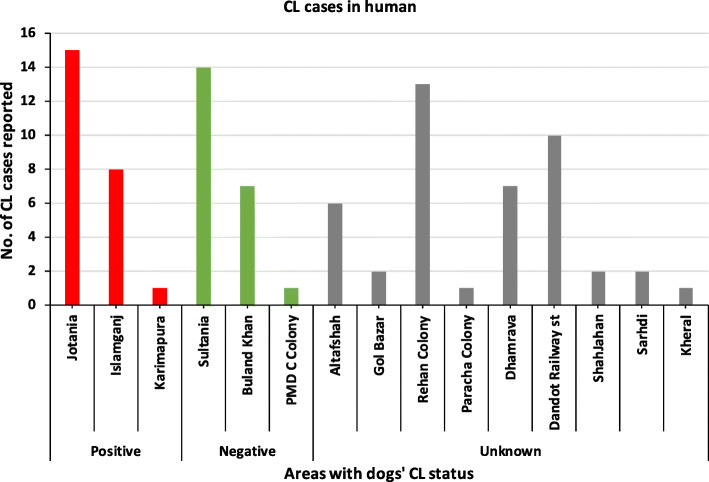
Number of human-CL cases according to areas and canine-CL cases

Among the cases, the individuals between the age group 1-15 years, had the greatest frequency (51%). The median of the age of human-CL patients was 15 (range: 1–76) (Fig. 2). The gender distribution was equal among cases (Male = 45; Female = 45). Most of the cases (*n* = 69, 76.7%) belonged to the income level category of PKRs 5000–10,000 (ranged from PKRs 5000-30,000). The hospital registration of human-CL patients was lower in winter months (December 2012-February-2013) and it peaked during summer and monsoon months (May–September 2013) followed by decline during October and November months (2013) (χ^2^ = 20.4, *p* < 0.05). The highest peak observed was in June and September 2013 (*n* = 12), whereas the lowest was in December 2012 and February 2013 (*n* = 2) (Fig. 3). The geographical locations of cases are marked on map in Fig. 4 (a) and showed that most of the cases were concentrated near Jotania (16.7%) and Sultania (16%) areas followed by Rehan Colony (Fig. 5). Locations of controls were not retrieved.

### Potential risk factors

A total of 180 individuals were contacted and interviewed (90 cases, 90 controls) from the study area. Ten variables were screened in univariable analysis and 8 were associated with being a case or control (Table 1). Poor sanitary conditions, presence of other *Leishmania* infected persons in the house, sleeping outsides in open areas, other animal on premises, and house type, gender (being female), using protections like insecticide sprays, bed nets, screens etc. and age (in years) were selected for multivariable analysis based on selection criteria (*p* < 0.025). Two variables ‘income of the participant’ and ‘keeping a dog’ having *p* > 0.25 were excluded from further analysis. One variable namely ‘other human-CL patient in house’ was excluded from analysis due to insufficient number of discordant pairs (Table 1).

**Table 1:** Risk factors for cutaneous leishmaniasis based on univariable analysis

Variables	Response	Case numbers (%)	Control numbers (%)	OR	95% CI	p-value
Age (years)	1–15	46 (51.1)	7 (7.9)	Ref		< 0.009^*!^
	15–30	27 (30)	40 (44.4)	0.11	0.03–0.34	
	30–45	11 (12.2)	29 32.2	0.07	0.02–0.25	
	45–60	5 (5.6)	12 (13.3)	0.08	0.02–0.35	
	> 60	1 (1.1)	2 (2.2)	0.02	0.01–3.26	
Sex	Male	45 (50)	18 (20)	0.25	0.12–0.52	0.0002^*^
	Female	45 (50)	72 (80)			
Income (PKRs)	5000–10,000	69 (76.7)	60 (66.7)	Ref		0.225
	10,000–20,000	16 (17.8)	21 (23.3)	0.64	0.29–1.44	
	20,000–30,000	4 (4.4)	8 (8.9)	0.46	0.13–1.59	
	> 30,000	1 (1.1)	1 (1.1)	0.68	0.37–12.26	
Dog in house	No	82 (91.1)	83 (92.2)	1.17	0.39–3.47	0.782
	Yes	8 (8.9)	7 (7.8)			
Other animals on premises	No	44 (48.8)	67 (74.4)	3.10	1.57–6.1	0.00114^*^
	Yes	46 (51.1)	23 (25.5)			
Poor sanitary conditions	No	16 17.8	43 47.8	4.37	2.03–9.43	<.00017^*^
	Yes	74 (80.2)	47 (52.2)			
Protection used	Yes	15 16.7	26 28.9	0.48	0.22–1.01	0.0535^*^
	No	75 (83.3)	64 (71.1)			
House type	Concrete	44 (48.9)	57 (63.3)	1.93	1.01–3.68	0.0461^*^
	Mud	46 (51.1)	33 (36.7)			
Other CL patients in house	No	37 (41.1)	86 (95.6)	50	6.91–361.9	0.000107^$^
	Yes	53 (58.9)	4 (4.4)			
Sleeping Outside in open area	No	6 (6.7)	62 (68.9)	12.2	4.90–30.36	0.0000056^*^
	Yes	84 (93.3)	28 (31.1)			

^*^Factors selected for multivariable analysis

^!^p-value bases on likelihood ratio test

^$^Excluded from analysis due to inadequate discordant pairs

In the final multivariable model, four variables were identified as significantly associated with the human-CL in Khewra residents (Table 2). Cases keeping other animals in house (livestock, poultry) were 3.6 times (95% CI: 1.07–12.12, *p* < 0.05) more likely to have human-CL compared to controls. Similarly, cases having poor sanitation conditions at home were more likely to have human-CL (OR: 3.3, 95% CI 1.03–10.56, *p *< 0.05) as compared to controls. The odds of being diagnosed with human-CL were 8.7 time more in cases who slept outside in open area (95% 2.90–26.37, *p* < 0.001) when compared to exposure in controls. The increasing age showed to have decreased the likelihood of human-CL 0.4 times (95% CI: 0.25–0.76, *p* < 0.005) (Table 2).

**Table 2:** Risk factors in final multivariable logistic model

Variables	Odds ratio	95% CI	p-value
Age (years)	0.4	0.25–0.76	0.003
Other animals in house	3.6	1.07–12.12	0.039
Poor sanitary conditions	3.3	1.03–10.56	0.045
Sleeping outside in open area	8.7	2.90–26.37	0.0001

### Prospective study in dogs

During the study period (January–April 2014), 15 blood samples of the dogs brought to Civil Veterinary Hospital with skin lesions were collected. A dry thin stained smear was made from the blood sample for the detection of amastigote forms of *Leishmania* in macrophages of the dogs. *Leishmania* amastigotes forms were found in 4 out of 15 samples (21%). The presence of positive dogs was significantly associated (*p* < 0.001) with the positive cases of human-CL in the same area. The spatial distribution of the canine-CL cases with human-CL cases is shown in Fig. 4 (b). The dogs from Jotania, Islamganj and Karimpura areas were tested positive for canine leishmaniasis (Fig. 5). The status of the dogs from other areas was unknown.

## Discussion

The epidemiological triad of CL is complex with various epidemiological risk factors associated with host, agent and environment. Early recognition of these risk factors may prevent the further transmission to susceptible population. Results of our study support the findings of other studies from Pakistan that CL cases are increasing in the local human and dog population in Pakistan [9, 12, 13, 30, 31] suggesting that a one-health approach would be needed to reduce the disease burden.

Published literature about risk factors for human-CL in Pakistan in generally sparse or obsolete [8, 32, 33]. The current study was aimed to determine the risk factors associated with human-CL that prevailed in the local environment and detection of canine-CL in dogs in Khewra region, District Jhelum. After extensive literature review, age, sex, income, keeping dog, keeping other animals (livestock, poultry), poor sanitary conditions, sleeping outside in open areas, using protections and presence of other *Leishmania* infected persons in the house were included as risk factors [2, 12, 30, 34–36].

Seasonal pattern of transmission is useful to establish disease surveillance and control activities [37]. Human-CL patients registered in the current study, visited the hospital over a period of 12 months. There was a significant increase in the patient visits to the hospital during summer and monsoon months (May–September 2013). This reflects seasonal activity of sand-fly vector during summer and rainy season. Jhelum experience monsoon season from June to September which brings heavy rain, while the dry season in this region is from November to January. Varied transmission patterns have been reported by various studies suggesting seasonal trends in different geographical locations [37–39].

Previous studies have reported clustering of leishmaniasis at household level [33, 38, 40]. In current study, among all cases, 59% (*n* = 53) confirmed the presence of other human-CL patient in their house or in the neighbors. Presence of an infected person in the household increases the risk of getting infected because sand-flies have limited fly zone and they remain in same vicinity and could bite multiple hosts living at the same place [32, 38, 40]. *L. tropica* transmission has also been known to be characterized by clustering of cases [32].

In the current study, we identified a set of risk factors that might significantly contribute to the web of causation of human-CL in the region. Age (increasing in years) was identified as a protective factor (OR < 1). The majority of the cases in our study were children and young people < 15 years of age and human-CL was less reported in adults compared to children age groups. Increasing each year in age reduced the likelihood of human-CL 0.4 times. This could be correlated to the outdoor activities of the children (playing outdoor games) with minimum precautions to cover their body, which might have exposed them to bites of sand-flies, while adults adopt more precautions during outdoor activity [38, 41, 42].

Keeping other animals in house (livestock, poultry) showed association with CL (OR > 1). Presence of other animals e.g. livestock and poultry, could attract vector of CL due to presence of barn and dried dung, and may expose residents to the female sand-flies. Previously, presence of sand-flies was reported to be associated with cattle and cattle blood was found in *Phlebotomus tobbi* females [41, 43, 44].

Patients reporting poor sanitary condition were 3.3 times more likely to diagnosed with human-CL compared to those with better condition. Almost 80% of the cases reported unsatisfactory sanitary condition at home, which included, open toilets, open sewage, mud floors and unhygienic livings. Poor sanitary conditions provide suitable habitat for sand-flies to breed and spread human-CL [45, 46]. Studies have consistently shown more cases of human-CL among poor, neglected populations, who are likely to be less educated and mostly unemployed [41, 47]. Furthermore, most of cases (76.7%) in current study, belonged to families having an income of PKRs 10,000 or less. Families with lower income level have less resources to adopt protective measure and awareness about the protections against diseases, consequently increased risk of exposure to sand-flies and *Leishmania* infection [2]. Interventions such as poverty alleviation and improving living condition might aid significantly in controlling human-CL transmission in the region [21].

Sleeping outside in open air increased the odds of human-CL, probably due to their exposure to the sand-fly bites during the sleeping time as one cannot protect oneself. Sleeping outdoor in open space during summer months is very common in Pakistan. Our findings corroborated with the results of other studies that sleeping outside is a risk factor for CL [3, 41, 47, 48]. In Pakistan, May–September are hot and humid months and people, especially in villages prefer to sleep outside the rooms in open air. Sand-fly activity is also increased through June and July, with peak in August. Entomological studies indicated nocturnal activity of sand-flies starts at the beginning of the night, and is strongly associated with relative humidity rather than with temperature [3, 41, 47]. Our data also supported the speculation that the most appropriate transmission period of CL is during the hot and humid nights from July to September.

During the period of 3 months, 15 suspected dogs for CL were brought to the local veterinary hospital. Their blood samples were taken and tested for presence of *Leishmania* protozoa. Among 15 suspected, 21% (*n* = 4) were detected positive for canine-CL. Although dogs are considered major reservoir for *L. infantum,* the possibility of clinical canine disease and their potential as secondary hosts for *L. major* should be investigated in endemic areas for human *L. major* infection [28, 34]. Our results showed a significant association between the areas of reported cases of human-CL and canine-CL positive dogs. Areas with high burden of human-CL cases had presence of canine-CL positive dogs.

The findings of the study have some limitations due to case-control nature of the design as it is difficult to establish the temporal causality in case-control studies. Furthermore, these study designs are prone to selection bias and recall bias. Future investigations based on cohort study design would be more appropriate to ascertain the causal relationship between risk factors and outcome.

## Conclusions

Pakistan has a diverse landscapes and climates that may affect the transmission of *Leishmania* in the country. The key risk factor identified in present study may be extrapolated to design an early preparedness response for human-CL outbreaks at human-animal interface. The current study also provides initial evidence for the presence of canine-CL in Khewra region, District Jhelum.

## Supplementary Information

**Additional file 1.**

## Data Availability

The data gathered and generated during the current study are available from the corresponding author (Mamoona Chaudhry) on reasonable request.
