# Supplementary material for: Cutaneous Leishmaniasis in Pakistan: a neglected disease needing one health strategy
Source: BMC Infect Dis. 2021 Jun 30;21:622. doi: 10.1186/s12879-021-06327-w (PMC8243581; doi:10.1186/s12879-021-06327-w)
Supplement: Supplementary file 1 — Additional file 1. [file 12879_2021_6327_MOESM1_ESM.docx]

**Questionnaire for case-control study of CL in human patients**

**Do you consent to participate in study:** Yes No

**GENERAL INFORMATION**

Name: Address:

Contact no.:

Date of registration to hospital: _______________

- Age: ___________ Years
- Sex: Male Female
- Income: 5000-10000 10000-20000 20000-30000 >30000

**EXPOSURE INFORMATION**

1. Do you keep a dog in/outside your house? Yes No
2. Do you keep other animals (livestock, poultry)? Yes No
3. Do you have poor sanitation in the house? Yes No
4. Do you use protection (insecticide spray, lotion, screen) against insects? Yes No
5. What kind of house you live in? Mud house Concrete house
6. Do you sleep outside in open area? Yes No
7. Is there any leishmaniasis patient in the house? Yes No
